# Modern management of BCG-refractory non-muscle-invasive urothelial carcinoma of the urinary bladder

**DOI:** 10.1007/s00120-025-02625-2

**Published:** 2025-06-23

**Authors:** Domenique Escobar, Chirag Doshi, Mazyar Zahir, Siamak Daneshmand

**Affiliations:** Catherine and Joseph Aresty Department of Urology, University of Southern California/Norris Comprehensive Cancer Center, University of Southern California, 1441 Eastlake Ave, Suite 7416, 90089 Los Angeles, CA USA

**Keywords:** Non-muscle-invasive bladder cancer, BCG-refractory, Intravesical, Gene therapy, Radical cystectomy, Nichtmuskelinvasives Blasenkarzinom, BCG-refraktär, Intravesikal, Gentherapie, Radikale Zystektomie

## Abstract

**Background:**

High-risk non-muscle-invasive bladder cancer (NMIBC) is most commonly treated with Bacillus Calmette–Guérin (BCG) as first-line therapy. However, in light of the ongoing BCG shortage in the United States, a significant need exists for alternative treatment options, both in the upfront setting as well as for patients with BCG-refractory disease. While radical cystectomy remains the gold standard for patients with BCG-refractory disease, many patients are unfit or unwilling to undergo this procedure. Several new agents, many with novel mechanisms of action, have been approved or are actively being investigated in this setting.

**Materials and methods:**

Articles were chosen for review based on expert knowledge of the literature as well as on PubMed literature searches for the topics of focus. Appropriate articles were selected for inclusion by reviewing article titles, abstracts, and full texts.

**Results:**

There is ample evidence for emerging therapies in BCG-refractory NMIBC including systemic immunotherapy and various intravesical options, such as chemotherapy, gene therapy, and novel drug delivery systems. Radical cystectomy, however, remains the gold standard. Response rates and duration of response vary across treatment modalities, with complete response rates at any time ranging from 41% to 82%. Radical cystectomy should still be recommended to patients with high-risk features given the risks of recurrence, progression, upstaging, and occult lymph node metastases.

**Conclusion:**

While radical cystectomy remains the standard of care for patients with BCG-refractory disease, many patients are unable or unwilling to undergo the procedure. Several novel therapies have been recently approved or are currently being investigated with overall promising early results.

## Abstract (de)

**Hintergrund:**

Die Hochrisikoform des nichtmuskelinvasiven Blasenkarzinoms (NMIBC) wird am häufigsten mit dem Bacillus Calmette-Guérin (BCG) als Erstlinientherapie behandelt. Angesichts des fortbestehenden Mangels an BCG in den USA besteht ein erheblicher Bedarf an alternativen Therapieoptionen, sowohl in der Erstbehandlung als auch für Patienten mit BCG-refraktärer Erkrankung. Zwar stellt die radikale Zystektomie immer noch den Goldstandard für Patienten mit BCG-refraktärer Erkrankung dar, aber viele Patienten sind nicht fit genug oder nicht dazu bereit, diese Behandlung mitzumachen. Zahlreiche neue Substanzen, viele mit neuartigen Wirkmechanismen, sind für diese Situation zugelassen worden oder werden derzeit aktiv untersucht.

**Material und Methode:**

Die Auswahl der Artikel für diese Arbeit erfolgte auf der Grundlagen von Expertenwissen hinsichtlich der Literatur sowie basierend auf Literaturrecherchen in PubMed in Bezug auf die Schwerpunktthemen. Geeignete Artikel wurden für den Einschluss in die Studie anhand der Durchsicht der Titel, der Zusammenfassungen und der Volltexte der Beiträge ausgewählt.

**Ergebnisse:**

Es gibt reichlich Evidenz für aufkommende Therapieansätze bei BCG-refraktärem NMIBC einschließlich systemischer Immuntherapie und verschiedener intravesikaler Optionen, wie Chemotherapie, Gentherapie und neuartige Medikamentenverabreichungssysteme. Die radikale Zystektomie stellt jedoch weiterhin den Goldstandard dar. Die Ansprechraten und die Dauer des Ansprechens variieren je nach Behandlungsmodalitäten, dabei reichen die Raten des vollständigen Therapieansprechens zu einem beliebigen Zeitpunkt von 41 bis 82%. Eine radikale Zystektomie sollte weiterhin bei Patienten mit Hochrisikomerkmalen angesichts des Risikos der Rezidivierung, Progression, der Transformation in ein höheres Tumorstadium und okkulter Lymphknotenmetastasen empfohlen werden.

**Schlussfolgerung:**

Zwar bleibt die radikale Zystektomie der Goldstandard in der Therapie von Patienten mit BCG-refraktärer Erkrankung, aber viele Patienten sind zur Durchführung dieser Behandlung nicht in der Lage oder nicht dazu bereit. Zahlreiche neuartige Substanzen sind vor Kurzem zugelassen worden oder werden derzeit mit insgesamt vielversprechenden ersten Ergebnissen untersucht.

## Introduction

Bladder cancer is a common malignancy in the United States, with over 83,000 new cases diagnosed in 2024 [1]. Non-muscle-invasive bladder cancer (NMIBC) comprises the majority of new bladder cancer diagnoses, and while prognosis is generally favorable, certain challenges remain, particularly in the management of Bacillus Calmette–Guérin (BCG)-refractory disease.

In light of the ongoing BCG shortage in the United States, BCG is typically reserved for patients with American Urological Association (AUA) high-risk disease, although its use may be considered in patients with intermediate-risk disease. According to the AUA, high-risk disease includes patients with high-grade (HG) T1, recurrent or multifocal HGTa, HGTa >3 cm, carcinoma in situ (CIS), variant histology, lymphovascular invasion, or HG prostatic urethral involvement (Table 1). Bacillus Calmette–Guérin is administered with an induction course (once a week for 6 weeks) and ideally followed by maintenance cycles for up to 3 years. The European Association of Urology (EAU) has similar risk stratifications with some important distinctions as well as further stratifications based on tumor grade (G1, G2, G3) and three primary risk factors: age >70, multiple papillary tumors, and tumor diameter >3 cm. In the EAU guidelines, a very high risk group exists that includes those with HGTa and HGT1 with or without CIS and varying numbers of risk factors. The high-risk group also includes HGT1 without CIS or isolated CIS except those who are included in the very high risk group, as well as LG and HG Ta/T1 without CIS and varying numbers of risk factors [2].

**Table 1:** American Urological Association risk categories in non-muscle-invasive bladder cancer

Low risk	Intermediate risk	High risk
Solitary LGTa ≤3 cm	Recurrence within 1 year of LGTa	HGT1
Papillary urothelial neoplasm of low malignant potential (PUNLMP)	Solitary LGTa >3 cm	Any recurrent HGTa
	Multifocal LGTa	HGTa >3 cm or multifocal
	HGTa ≤3 cm	Any CIS
	LGT1	Any variant histology
		Any LVI
		Any HG prostatic urethral involvement
		Any BCG failure in HG patient

*LG* low grade, *HG* high grade, *CIS* carcinoma in situ, *LVI* lymphovascular invasion

Recurrence of NMIBC is quite common with rates varying by cancer grade and stage. Progression to more advanced stages can also occur, particularly in high-grade or invasive tumors. Patients with HGT1 are at the highest risk, with 5‑year recurrence rates of over 40% and 5‑year progression rates of over 20%. By contrast, although patients with low-grade tumors are prone to recurrence, progression is much less common [3].

While patients treated with intravesical BCG generally have favorable outcomes with decreased risk of recurrence and progression [4, 5], failure is not uncommon and presents a challenge in the management of these patients. A prognostic analysis of patients from two European Organization for Research and Treatment of Cancer randomized phase 3 trials in intermediate- and high-risk NMIBC treated with BCG also highlighted the heterogeneous prognosis in this group. Overall, 1‑and 5‑year recurrence rates were 25.9% and 41.3%, respectively. Factors such as prior recurrences, tumor grade, and number of tumors impacted the probability of early recurrence, whereas tumor grade and T stage were significant predictors of progression [6].

In 2018, the US Food and Drug Administration (FDA) issued guidance for the definition of BCG-unresponsive or BCG-refractory disease in order to guide clinical trials in this area ([7]; Table 2). The following clinical scenarios comprise BCG-unresponsive or BCG-refractory disease:

- HGT1 at the first (3 month) evaluation following BCG induction
- Persistent/recurrent HGTa/CIS within 6 months of adequate BCG
- Recurrent HGTa/T1 within 6 months of adequate BCG
- Recurrent CIS within 12 months of the last adequate BCG treatment

**Table 2:** Definition of carious BCG disease states

BCG unresponsive or BCG refractory (terms commonly used interchangeably)	– HGT1 at the first (3 month) evaluation following BCG induction – Persistent or recurrent HGTa/CIS within 6 months of adequate BCG – Recurrent HGTa/T1 within 6 months of adequate BCG – Recurrent CIS within 12 months of last adequate BCG
BCG exposed	– HGTa/CIS 3 months after initiating BCG – Any late relapse (12–24 months after initiating BCG) of high-risk disease – Does not meet definition of BCG unresponsive
BCG relapse	– HG recurrence after reaching a disease-free state within 6 months of receiving adequate BCG

Adequate BCG includes at least five to six doses of induction plus either two to three doses of maintenance or five to six doses of a re-induction course.

Owing to the significant demand in the realm of BCG-refractory disease, new treatments and clinical trials are being developed and this area is rapidly evolving. In this article, we review the management of BCG-refractory NMIBC in the modern era including the role of repeat transurethral resection and provide a brief overview of therapies that are no longer recommended as well as a review of currently utilized treatments that have been recently approved or are being actively investigated. We also review the evidence for radical cystectomy in this population, which remains the gold standard.

## Methods

Articles were chosen for review based on expert knowledge of the literature as well as PubMed literature searches for the topics of focus. Appropriate articles were selected for inclusion by reviewing article titles, abstracts, and full texts. Particular attention was paid to recently published articles, highly anticipated scientific conference presentations, and landmark studies. Pertinent articles found from the references of the previously identified articles were also included when relevant.

## Results

### Role of repeat transurethral resection

A 2018 systematic review sought to assess the utilization and benefit of repeat transurethral resection (reTUR) in over 8400 patients with NMIBC. The proportion of cases with detrusor muscle at initial TUR varied greatly from 30% to 100%. Residual tumor at reTUR was not uncommon, ranging from 17% to 67% in patients with initial Ta tumors and from 20% to 71% in patents with initial T1 tumors. Rates of upstaging similarly varied, with 0–8% of patients with Ta upstaged to T1 or greater and 0–32% of patients with T1 upstaged to T2 or greater. Regarding recurrences, in those with initial Ta, 16% of patients who underwent reTUR experienced recurrences at 1 year compared to 58% in those who did not. In patients with initial T1, there was less of a distinction between those who did and did not undergo reTUR: 18–56% of those who underwent reTUR experienced a recurrence compared to 30–71% of those who did not. Regarding progression, in patients with initial Ta, rates were 7–13% in those who underwent reTUR compared to 31% in those who did not. Interestingly, in patients with initial T1 disease, 21% who underwent reTUR progressed, compared to 17% in those who did not. In those with initial T1, effects of reTUR on cancer-specific mortality were mixed but there was a modest reduction in overall mortality in those who underwent reTUR [8]. Nonetheless, reTUR remains a standard and important part of the management of patients with HGT1 disease particularly when no muscle is present in the initial specimen. In our practice, we do not routinely perform reTUR in cases of focal HGT1 if muscle was present in the initial specimen.

### Historical intravesical therapies

Valrubicin, an intravesical chemotherapy agent, was historically used as single-agent therapy in the treatment of BCG-refractory disease. Today, its use has generally fallen out of favor due to low durable response rates. In fact, single-agent valrubicin (and other single-agent chemotherapy medications) has been removed as a recommended agent for BCG-refractory disease in the most recent amendment to the AUA NMIBC guidelines. However, a recent study evaluated the use of combination valrubicin/docetaxel in a cohort of 75 patients who had been heavily pre-treated with BCG, gemcitabine/docetaxel, or both. The authors found promising results with 2‑year recurrence-free survival rates of 73% and 38% in patients with low-grade and recurrent high-grade disease, respectively. In patients with high-grade disease, overall, cancer-specific and cystectomy-free survival rates at 2 years were also excellent [9]. These findings indicate that combination valrubicin/docetaxel may be a reasonable option in previously treated patients who are unwilling or unable to undergo cystectomy. Thiotepa, another historically used intravesical chemotherapy agent, is no longer recommended due to side effects and the development of more efficacious medications. There are several other agents that have been studied that are not routinely used today such as vicinium, *Mycobacterium phlei *cell wall-nucleic acid complex (MCNA), BCG plus interferon, Nab-paclitaxel, and paclitaxel-hyaluronic acid, among others [10].

### Overview of emerging therapies

A 2020 systematic review evaluated 30 studies on current and emerging therapies used to treat patients who had received BCG, with the majority being classified as BCG unresponsive or refractory. There was significant variation in endpoint definitions, assessment timepoints, population characteristics, and inclusion criteria among the included studies, highlighting the importance of establishing clear definitions and guidelines for studies in this setting. At the time this study was published, valrubicin was the only approved agent for BCG-refractory disease with a mere ~10% complete response rate at 12 months. However, many of the other included studies assessed agents that will be discussed in more depth below. These comprise agents with novel mechanisms including gene therapy with production of interferon alfa-2b (nadofaragene firadenovec or Adstiladrin®), oncolytic immunotherapy (cretostimogene grenadenorepvec or CG-0070), and viral gene therapy inducing proliferation of natural killer and T cells (Anktiva®; [10]).

### Systemic immunotherapy

Pembrolizumab, a PD‑1 inhibitor, is a form of systemic immunotherapy that was approved by the FDA in 2020 for patients with BCG-unresponsive, high-risk NMIBC with CIS with or without papillary tumors who are unfit for or who decline cystectomy. In the KEYNOTE-057 study, which included 96 patients with BCG-unresponsive disease, the complete response rate at 3 months was 41%. Of these responders, 46% (or 18% of the overall cohort) remained in complete response for 12 months or longer. Additional data have shown that only 11% of the overall cohort remained in complete response at 18 months. Adverse events from treatment were also not uncommon—13% of patients in the study experienced grade III or IV adverse events (most commonly arthralgia and hyponatremia) and serious adverse effects occurred in 8% of patients [11]. This agent is not widely used in this setting given these adverse events and the availability of intravesical therapies with lower toxicity profiles and better response rates.

### Intravesical therapies

#### Gemcitabine/docetaxel

Intravesical combination chemotherapy with gemcitabine and docetaxel (gem/doce) is a commonly used regimen in BCG-refractory disease. Multi-institutional data have shown that 1‑ and 2‑year recurrence-free survival rates were 60% and 46%, and high-grade recurrence-free survival rates were 65% and 52%, respectively [12]. Given the ongoing BCG shortage, this regimen is also being used as first-line therapy. In a study of 107 patients with BCG-naïve high-risk NMIBC receiving gem/doce, who were followed up for a median of 15 months, recurrence-free survival was 89% at 6 months, 85% at 12 months, and 82% at 24 months [13]. These findings have prompted the development of the BRIDGE trial (a Randomized Phase 3 Trial of Intravesical BCG versus Intravesical Docetaxel and Gemcitabine Treatment in BCG Naïve Non-Muscle Invasive Bladder Cancer, NCT05538663) for a head-to-head comparison of BCG and gem/doce. This trial is nearly completely accrued and will provide important information about whether gem/doce is an equally efficacious alternative to BCG in the treatment of these patients. Importantly, gem/doce also has an excellent financial profile, particularly when compared with the novel agents discussed below, which tend to be quite expensive.

Of note, there are also data supporting the use of gemcitabine monotherapy in BCG-refractory high-risk NMIBC. A prospective, randomized, phase 2 trial assessed gemcitabine versus repeat BCG in this patient group and found no difference in mean time to first recurrence but significantly higher 2‑year recurrence-free survival in the gemcitabine group compared to the BCG group (19% vs. 3%; [14]). However, in our practice we favor doublet therapy with gem/doce.

#### Adstiladrin®

Adstiladrin®, or nadofaragene firadenovec, is one of the newest intravesical agents approved in 2022 by the FDA for use in patients with BCG-unresponsive NMIBC, specifically CIS with or without papillary tumors. Adstiladrin® was the first gene therapy approved in this space. It is a recombinant, non-replicating adenovirus serotype 5 vector containing DNA that encodes interferon alfa-2b (IFNα2b), which has established anticancer effects. It is administered intravesically once every 3 months. However, this agent should be avoided in patients who are immunocompromised due to the risk of disseminated adenoviral infection. Phase 3 clinical trial data reported a 53.4% complete response rate at 3 months after the first dose, and 45.5% of the complete responders continued to have a complete response at 1 year; 25% of patients maintained a complete response at 36 months [15, 16]. The 5‑year follow up demonstrated high-grade recurrence-free survival rates of 13–33% and cystectomy-free survival rates of 49–59% at 60 months. Only five patients progressed to muscle-invasive disease [17]. The medication was also very well tolerated with only 4% of patients experiencing grade III adverse events. This area continues to evolve with ongoing studies investigating its use in various settings as part of the ABLE (Adstiladrin in BLadder CancEr) clinical trial program. For example, the ABLE-22 study (NCT06545955) is a phase 2 trial evaluating the use of Adstiladrin® alone or in combination with chemotherapy (gemcitabine or docetaxel) or immunotherapy (pembrolizumab) in BCG-unresponsive CIS ± Ta/T1. The ABLE-32 study (NCT06510374) is a randomized control trial comparing Adstiladrin® with observation in patients with intermediate-risk disease and the ABLE-41 registry study (NCT06026332) is evaluating real-world experience with its use.

#### Anktiva®

Anktiva®, also known as nogapendekin alfa inbakicept-pmln or N‑80 ± 3, is another type of gene therapy. It is an IL-15 superagonist that induces the proliferation of natural killer and T cells. This agent was approved by the FDA in 2024 for BCG-unresponsive disease with CIS with or without papillary tumors. In a study with 160 patients, when given in combination with BCG, the complete response rate in patients with CIS with or without papillary disease was 71% with a median duration of 27 months. Patients with papillary-only disease had disease-free rates of 57% at 12 months and 48% at 24 months. However, it is important to note that the efficacy of Anktiva® is dependent on the concomitant administration of BCG, as evidenced by the closure of the monotherapy arm in the original trial. Due to this, its utilization may be hindered by the national shortage of BCG in the United States, but efforts are underway to provide recombinant BCG to be used with the drug to alleviate this issue. The medication was also well tolerated, but 20% of patients experienced grade III complications or higher, which is notably higher than with Adstiladrin®, for example [18, 19].

#### Agents in development and clinical trials

Due to the significant need for additional treatments for patients with BCG-refractory disease, several new agents have been developed, with fast-track designation from the FDA, and numerous clinical trials are ongoing.

**Fig. 1:**
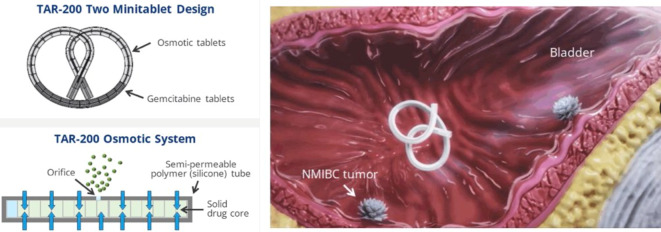
TAR-200 intravesical drug delivery system for sustained release of gemcitabine. *NMIBC* non-muscle-invasive bladder cancer. (Source: Johnson & Johnson ©)

TAR-200 is a novel intravesical drug delivery system that allows for a sustained release of gemcitabine in the bladder, increasing dwell time (Fig. 1). It is generally well tolerated with few significant side effects or symptoms and has shown to be effective in the neoadjuvant setting in patients with muscle-invasive bladder cancer [20]. The SunRISe‑1 randomized clinical trial (NCT04640623) is ongoing and is evaluating TAR-200 ± cetrelimab and cetrelimab alone in patients with BCG-refractory high-risk NMIBC (CIS ± papillary disease) who are unfit for or who decline cystectomy. In the TAR-200 monotherapy group, complete response rates were found to be as high as 84% at any time, with 82% of patients maintaining the response after a median follow-up of 9 months. The estimated 12-month complete response rate in this group is 57%. Importantly, it does not appear that there are any synergistic effects with checkpoint inhibitors, as the complete response rates are essentially the same between the monotherapy and combination groups. In addition, very few patients discontinue treatment secondary to adverse effects (< 5%; [21]). TAR-210 is another intravesical drug delivery system for erdafitinib, an FGFR inhibitor. It has shown promise, with a recurrence-free rate of 82% in high-risk patients and complete response rate of 87% in intermediate-risk patients [22].

Cretostimogene grenadenorepvec (CG-0070) is an intravesical, adenovirus-based oncolytic vaccine that is designed to replicate selectively in tumor cells with alterations in the retinoblastoma pathway. In addition, it has an immunotherapeutic mechanism of action by promoting expression of GM-CSF, which in turn has anti-tumor effects. The BOND-003 trial (NCT04452591) evaluated 112 patients with high-risk BCG-unresponsive NMIBC receiving this agent. The medication was administered with six weekly doses during induction, followed by three weekly maintenance cycles at months 3, 6, 9, 12, and 18. The complete response rate at any time was found to be 75%, and 83% of responders had an ongoing response at 12 months. It was also generally well tolerated [23]. The CORE-008 trial (NCT06567743) is an ongoing phase 2 clinical trial studying the use of this agent in patients with high-risk NMIBC. There are two cohorts in the trial: Cohort A (BCG-naïve) includes patients with CIS ± HG Ta/T1 who have not received prior BCG, and Cohort B (BCG-exposed) will consist of patients with CIS ± HG Ta/T1 or papillary-only disease who have received prior BCG and have experience recurrence either immediately after induction therapy (BCG-resistant) or at a delayed timepoint, after adequate or inadequate BCG [24].

The LEGEND study (NCT04752722) is a phase 1/2 study of detalimogene voraplasmid (EG-70), a non-viral intravesical gene therapy for patients with BCG-refractory NMIBC with CIS ± papillary tumors who are unfit for or who decline cystectomy. Preliminary results from 24 patients in the phase 1 portion of the trial found that the overall complete response rate was 73%, 68% at 3 months, and 45% at 6 months. Among responders, 73% and 60% had durations of response of ≥3 months and ≥6 months, respectively. Roughly 50% of patients experienced an adverse event, mainly grade I or II. The multi-cohort phase 2 portion is ongoing and is evaluating the efficacy of EG-70 in patients with BCG-naïve, exposed, or unresponsive disease with CIS and in those with BCG-naïve with CIS and BCG-unresponsive disease with high-grade papillary disease without CIS. The recommended dosing and schedule for phase 2 of the trial was established as four instillations of EG-70 0.8 mg/mL in a 12-week cycle. [25].

TARA-002 is another novel and promising option that is actively under investigation. This agent is a broad-spectrum immune potentiator that induces a TH1 pro-inflammatory cytokine response. It is a lyophilized biological preparation for instillation containing *Streptococcus pyogenes* (Group A, type 3) strain cells treated with benzylpenicillin. The ADVANCED-2 trial (NCT05951179) is evaluating patients with both BCG-refractory disease as well as BCG-naïve high-risk NMIBC (CIS ± papillary tumors). Treatment includes induction (six weekly instillations) and maintenance (three weekly instillations up to 18 months). Preliminary results from 24 enrolled patients showed a 70% overall complete response rate in high-grade disease (80% for BCG-refractory patients and 67% for BCG-naïve patients). At 6 months, high-grade complete response rates were 72% overall (100% for BCG-refractory patients and 64% for BCG-naïve patients). All nine patients who had a complete response at 3 months maintained the complete response at 6 months. The agent also appeared to be well-tolerated with no serious adverse events [26].

### Radical cystectomy

While several new agents are in development and approved for use in BCG-refractory disease, radical cystectomy remains the gold standard in the management of these patients. In a recent multi-institutional study of 578 patients with BCG-refractory disease treated with cystectomy versus bladder-sparing therapies, rates of recurrence and progression to muscle-invasive disease were found to increase over time with bladder-sparing therapy. While initial outcomes were similar, progression to MIBC was seen in 7% and 13% of patients treated with bladder-sparing therapies at 12 and 24 months, respectively. In addition, cystectomy was ultimately performed in 32% of patients who initially received bladder-sparing therapies, and nodal disease was found in 13% of these patients compared with 4% in those who received upfront cystectomy [27].

As previously discussed, patients with HGT1 disease are at the highest risk of recurrence and progression among all the types of NMIBC. One study found progression probabilities of 11.4% and 19.8% at 1 and 5 years, respectively, and probability of death due to bladder cancer of 4.8% and 11.3% at 1 and 5 years, respectively, in patients with HGT1 disease [6]. In addition, roughly 10–50% of patients will be upstaged to muscle-invasive disease on repeat transurethral resection, depending on whether muscle was present on the initial resection. However, this is a heterogeneous group of patients and management can be variable. Patients with BCG-refractory HGT1 should be strongly advised to undergo radical cystectomy. In this setting, and also in the upfront setting, cystectomy should be strongly considered if there is persistent HGT1 at re-resection, concomitant CIS, prostatic urethral involvement, variant histology, multifocality or tumor size >3 cm, lymphovascular invasion, hydronephrosis, or deeper invasion of the lamina propria, as these factors are associated with progression and death. Cure rates are excellent after cystectomy for HGT1, with cancer-specific survival shown to be ~65–93% in multiple studies. But it is important to note that upstaging (ranging from 26% to 51% in multiple studies) and even occult lymph node metastasis (ranging from 7% to 15% in multiple studies) at cystectomy is not uncommon in patients clinically deemed to be T1. These findings also highlight that challenges remain in accurately staging these patients. In addition, survival rates have been shown to be poorer in those undergoing delayed cystectomy compared to those undergoing early cystectomy [28]. Another study found that in patients with BCG-refractory NMIBC, renal impairment and tumor size >3 cm were independent predictors of progression-free survival and overall survival. Tumor size was also an independent poor prognostic factor for cancer-specific survival, whereas the presence of variant histology was a poor prognostic factor for overall survival. The authors concluded that patients with these risk factors should be strongly considered for cystectomy. Those who did not undergo cystectomy had significantly worse progression-free survival, cancer-specific survival, and overall survival compared to those who did [29].

Given this compelling evidence, the recommendation for radical cystectomy (upfront in certain high-risk patients and in BCG-refractory disease) are also reflected in various guidelines, including the AUA/Society of Urologic Oncology [30], National Comprehensive Cancer Network (NCCN; [31]), and EAU [2].

Lastly, while beyond the scope of this article, it is important to note that the presence of variant histology portends a worse prognosis with high rates of progression and upstaging. Upfront cystectomy should be strongly considered in these patients [32], especially in view of the fact that the data on the response of variant histology tumors to intravesical therapy are mixed.

## Discussion

The management of patients with BCG-refractory NMIBC is a field that is rapidly evolving. Several new therapies are already approved in the United States, and many clinical trials on other novel agents are underway (Table 3).

**Table 3:** Summary of novel and emerging therapies in BCG-refractory non-muscle-invasive bladder cancer

Type of therapy	Name of agent	Efficacy key points
Systemic immunotherapy	Pembrolizumab	*KEYNOTE-057* 41% CR at 3 months, 46% of responders maintained CR at 12 months or longer
Intravesical chemotherapy	Gemcitabine/Docetaxel	60% and 46% RFS at 12 and 24 months in BCG-refractory patients; 82–89% at 6–24 months in BCG-naïve patients
Intravesical gene therapy	Adstiladrin®	53.4% CR rate at 3 months, 45.5% of responders continued to have CR at 12 months 25% of patients maintained CR at 36 months
	Anktiva®	In CIS patients, CR rate 71% with median duration of 27 months. In papillary disease patients, disease-free rates of 57% at 12 months and 48% at 24 months
	Detalimogene voraplasmid (EG-70)	*LEGEND Trial* Overall CR rate was 73%, 68% at 3 months, and 45% at 6 months. Among responders, 73% and 60% had durations of response ≥3 months and ≥6 months
Intravesical drug delivery system	TAR-200 (Gemcitabine)	*SunRISe‑1 Trial* CR rates up to 84% at any time with 82% of patients maintaining CR after median follow-up of 9 months. Estimated 12-CR rate is 57%
	TAR-210 (Erdafitinib)	Investigations underway; preliminary results with recurrence-free rate of 82% in high-risk patients
Adenovirus-based oncolytic vaccine	Cretostimogene grenadenorepvec (CG-0070)	*BOND-003 Trial* CR rate at any time was 75% and 83% of responders had an ongoing response at 12 months
Immune potentiator	TARA-002 (lyophilized biological preparation containing cells of Streptococcus pyogenes (Group A, type 3) treated with benzylpenicillin)	*ADVANCED‑2 Trial* 70% overall HG CR rate (80% for BCG-refractory patients, 67% for BCG-naïve patients) At 6 months, HG CR rates were 72% overall (100% for BCG-refractory patients and 64% for BCG-naïve patients)

*CR* complete response, *RFS* recurrence-free survival, *HG* high grade

The current options for treatment of these patients are broad and include systemic immunotherapy, various intravesical options (including chemotherapy, gene therapy, novel drug delivery systems), and radical cystectomy, which remains the gold standard. Response rates vary greatly across modalities. The use of systemic immunotherapy has limited long-term efficacy with relatively high rates of adverse events. The use of intravesical chemotherapy with gemcitabine and docetaxel is well tolerated and effective but currently lacks prospective data (although this is actively being investigated in the BRIDGE clinical trial). Novel gene therapy agents, Adstiladrin® and Anktiva®, have shown promise in terms of efficacy and represent the first agents with this novel mechanism of action. Adstiladrin® has a favorable dosing schedule, which helps reduce the burden on patients. Anktiva® similarly has shown promise but relies on BCG, which is an ongoing challenge. Other novel agents such as cretostimogene and TAR-200 have also been given FDA fast-track designation.

Within NMIBC, patients with HGT1 disease represent the group with the highest risk of recurrence (~20–40% at 5 years), progression (~20% at 5 years), upstaging at re-TUR or cystectomy and occult lymph node metastases at cystectomy (~7–15%; [3, 28]). When high-risk factors such as the presence of variant histology or concomitant CIS are present, these rates are expected to increase. While many patients may desire bladder preservation and options for this are growing, it is important to counsel patients on the risks of progression and recurrence with bladder-sparing therapies (particularly with each successive therapy) and the benefit in oncologic outcomes that early radical cystectomy confers. This “window” in which a patient can be reliably cured prior to progression to more advanced disease cannot be overstated, particularly in those with the aforementioned risk factors.

## Conclusion

Management of Bacillus Calmette–Guérin-refractory non-muscle-invasive bladder cancer in the modern era is rapidly evolving and there are more options than ever to treat these patients, with promising new agents on the horizon.

## Declarations

This article does not contain any studies with human participants or animals performed by any of the authors.
